# DNA Copy Number Analysis in Gastrointestinal Stromal Tumors Using Gene Expression Microarrays

**DOI:** 10.4137/cin.s387

**Published:** 2008-03-27

**Authors:** Cristina R. Antonescu, Kai Wu, Guoliang Leon Xing, Manqiu Cao, Yaron Turpaz, Margaret A. Leversha, Earl Hubbell, Robert G. Maki, C. Garrett Miyada, Raji Pillai

**Affiliations:** 1 Department of Pathology, Memorial Sloan-Kettering Cancer Center, New York, NY; 2 Affymetrix Inc., Santa Clara, CA; 3 Molecular Cytogenetics Core Facility, Memorial Sloan-Kettering Cancer Center, New York, NY; 4 Department of Medicine, Memorial Sloan-Kettering Cancer Center, New York, NY

**Keywords:** copy number change, gene expression, array-based comparative genomic hybridization, whole genome amplification, GIST, sarcoma

## Abstract

We report a method, *Expression-Microarray Copy Number Analysis* (ECNA) for the detection of copy number changes using Affymetrix Human Genome U133 Plus 2.0 arrays, starting with as little as 5 ng input genomic DNA. An analytical approach was developed using DNA isolated from cell lines containing various X-chromosome numbers, and validated with DNA from cell lines with defined deletions and amplifications in other chromosomal locations. We applied this method to examine the copy number changes in DNA from 5 frozen gastrointestinal stromal tumors (GIST). We detected known copy number aberrations consistent with previously published results using conventional or BAC-array CGH, as well as novel changes in GIST tumors. These changes were concordant with results from Affymetrix 100K human SNP mapping arrays. Gene expression data for these GIST samples had previously been generated on U133A arrays, allowing us to explore correlations between chromosomal copy number and RNA expression levels. One of the novel aberrations identified in the GIST samples, a previously unreported gain on 1q21.1 containing the *PEX11B* gene, was confirmed in this study by FISH and was also shown to have significant differences in expression pattern when compared to a control sample. In summary, we have demonstrated the use of gene expression microarrays for the detection of genomic copy number aberrations in tumor samples. This method may be used to study copy number changes in other species for which RNA expression arrays are available, e.g. other mammals, plants, etc., and for which SNPs have not yet been mapped.

## Introduction

Chromosomal aberrations are frequently observed in cancer, and whole-genome analysis of copy number change in tumor cells has become a useful tool for tumor classification, tumor marker discovery, and for studying tumorigenesis. The initial application of chromosomal comparative genomic hybridization (CGH), co-hybridizing differentially labeled tumor and normal genomic DNA to normal metaphase spreads, identified genomic regions of deletions and amplifications in various tumor samples and cell lines (1), allowing copy number estimation at around 10 megabase resolution. Recent advances in microarray technology have provided higher resolution tools for genome wide analysis of copy number estimations. An early array-based study used a spotted chromosome-specific library or cloned genomic fragments to investigate copy number changes in tumor samples (2). Later developments, using micro-arrays derived from genomic clones (3), cDNA (4), BAC clones (5) and oligonucleotides (6–12) provided higher resolution analyses. By using high-density SNP oligonucleotide microarrays, Bignell et al. (8) described an assay and algorithm for copy number analysis on various cancer cell lines to identify homozygous deletions and high-level amplification. Other oligonucleotide-based microarray studies used longer oligos, 60- or 70-mers, to identify copy number changes in cancer cells (6, 7).

Gene expression profiles have been used successfully to classify tumors (13), (14), including gastrointestinal stromal tumors (15). To better understand the role of DNA copy number aberration in tumorigenesis, efforts have been made to correlate gene expression patterns to specific genomic alterations (16–22). While genes in the altered genomic regions are not necessarily regulated by DNA dosage, copy number aberrations may influence genome-wide gene expression patterns. If both genomic DNA and RNA are available from the same sample, both copy number analysis and RNA expression analysis can be performed on the same arrays. Thus, it is possible to assess whether a probeset is in a region that is both amplified and over-expressed. Such regions may be of greater interest for further study, both to understand the pathogenesis of disease and to explore the possibility of discovering diagnostic biomarkers.

Gastrointestinal stromal tumor (GIST) is the most common mesenchymal tumor of the intestinal tract (23). GISTs express KIT protein and show in a significant number of cases activating mutations in either *KIT* or *PDGFRA* genes, encoding for class III tyrosine kinase receptors (24, 25). Cytogenetically, GISTs show a rather simple karyotype with common losses of chromosome 14, 22 and 1p, in most cases, regardless of the *KIT* genotype. Since these simple genomic copy number aberrations have been previously confirmed by metaphase CGH (26–28) and BAC-array analysis (29), GIST represents an ideal tumor model for evaluating array-based methods for copy number analysis.

In this study, we describe an assay for detecting copy number changes by hybridizing genomic DNA to oligonucleotide microarrays designed for RNA expression profiling. We applied this approach to examine the genomic copy number changes among various cell lines and GIST tumors. Algorithm and method development were performed on cell lines containing various numbers of X chromosomes and known deletions and amplifications. This method, Expression-Microarray Copy Number Analysis (ECNA), allowed us to readily identify genes that showed copy number alterations starting with as little as 5 ng genomic DNA. ECNA was validated on GIST tumors in which previously described as well as novel copy number aberrations were identified.

## Materials and Methods

### Cell lines and DNA

DNA samples used in this study fall into 3 categories: DNA extracted from cell lines, normal human blood, and GIST tumors. DNAs from cell lines containing different copy numbers for the X chromosome: 1X(NA01723A), 2X(NA09899), 3X(NA04626), 4X(NA01416), and 5X(NA06061) chromosomes and from a Chromosome 4 deletion cell line (NA04126) were purchased from the National Institute of General Medical Sciences (NIGMS) Human Genetic Cell Repository, Coriell Institute for Medical Research (Camden, NJ). A human breast cancer cell line, SK-BR-3, was obtained from the American Type Culture Collection (ATCC, Manassas, VA). DNA was extracted from the cultured cells using the DNA Maxi Kit (Qiagen, Inc., Valencia, CA). DNA from normal blood was obtained from AllCells, LLC (Emeryville, CA). GIST sample DNA was obtained using a standard organic phenol-chloroform procedure. There were 5 GIST samples from 4 patients. Three of the samples were taken from the primary tumor resection, and in one patient two abdominal recurrences removed at different time points were analyzed (GIST#159, 199). The diagnosis was confirmed by pathologic review and immunoreactivity for KIT. Three samples had a *KIT* exon 11 mutation (GIST# 159, 198, 199) and 2 had a *PDGFRA* exon 18 deletion (GIST#171, 204). All samples used for ECNA are listed in Supplementary Table 1.

### Whole genome amplifications, purification, fragmentation and labeling

5–25ng genomic DNA was amplified using QIA-GEN’s REPLI-g® kit (Qiagen) for 16 hours at 30 °C, according to the protocol provided by the manufacturer. Reaction volumes were between 150 and 200 μL (2–5 μg/μL yield). DNA from GIST tumors has been previously used for reliable genotyping results. The amplification products were purified by Qiagen genomic-tip (Qiagen) and quantified using a NanoDrop® spectrophotometer (NanoDrop Technologies, Wilmington, DE) at 260 nm. Fragmentation of purified DNA samples (100 μg) was carried out by adding 0.2 Unit of DNAase I (DNA Fragmentation Reagent, Affymetrix, Inc.) in 1X of Fragmentation Buffer (Affymetrix, Inc.), then incubated at 37 °C for 30 min. The fragmentation reaction was terminated by incubation at 95 °C for 10 min. The fragmentation products were then terminally biotinylated with DNA Labeling Reagent (Affymetrix, Inc.) at 37 ºC for 5 hours. The labeled fragments were then concentrated on YM-3 Microcon columns (Millipore, Billerica, MA).

### DNA hybridization, wash, staining and scanning

Labeled DNA fragments (0.5 μg/μl) were added in a hybridization mix containing: 1X HYB Mix, 2.5X Denhardt’s Solution (Sigma, St. Louis, MO), 0.125 μg/μl human Cot-1 DNA (Roche, Basel, Switzerland), 0.06 nM Oligo B2. (Affymetrix) and 10% DMSO (Sigma). The hybridization mix was heated at 95 °C for 5 min, followed by immediate cooling, and then hybridized to the GeneChip® Human Genome U133 Plus 2.0 arrays (Affymetrix) at 48 °C for 16 hours. After hybridization, arrays were washed with 3M TMACl in 0.4 × SSPE and 0.01% Tween-20 solution for 30 min, then washed extensively with 0.1M NaCl in 0.6 × SSPE and 0.01% Tween-20 prior to the staining. Arrays were first stained with streptavidin, and then with a biotin-conjugated anti-streptavidin antibody, finally followed by staining with phycoerythrin—streptavidin. Arrays were scanned using the Affymetrix GeneChip Scanner 3000 (Affymetrix). Image analysis was performed with GeneChip Software GCOS, version 1.2.

### RNA hybridization to Affymetrix U133A arrays

RNA from 10 tumor samples (4 GISTs and 6 leiomyosarcomas) was analyzed on Affymetrix human genome U133A arrays. Leiomyosarcomas, which are malignant mesenchymal neoplasms of smooth muscle derivation closely resembling GIST morphologically, but genetically distinct from GIST, were used as a control reference. RNA was isolated using the protocol accompanying the RNAwiz™ RNA Isolation Reagent from Ambion (Austin, TX) and all samples were treated on the column with RNase-free DNase (Qiagen, Valencia, CA) according to the manufacturer’s instructions. Twenty-five to 50 nanograms of total RNA were tested for quality on an RNA 6000 Nano Assay (Agilent, Palo Alto, CA) using a Bioanalyzer 2100. RNA with an OD260/280 ratio greater than 1.8 was chosen for expression profiling experiments. Two micrograms of high quality total RNA was then labeled according to protocols recommended by the manufacturer. Briefly, after reverse-transcription with an oligo-dT-T7 (Genset), double stranded cDNA was generated with the Superscript double stranded cDNA synthesis custom kit (Invitrogen Life Technologies, Carlsbad, CA). In an in vitro transcription step with T7 RNA polymerase (MessageAmp™ RNA kit from Ambion) the cDNA was linearly amplified and labeled with biotinylated nucleotides. Ten micrograms of labeled and fragmented cRNA were then hybridized onto a test array and a Human Genome U133A expression array (Affymetrix, containing probesets representing 18,000 transcripts and variants). Post hybridization staining and washing were processed according to the manufacturer (Affymetrix). Finally, chips were scanned with a GC3000 laser confocal scanner.

### Data analysis of copy number change

**Figure 1:**
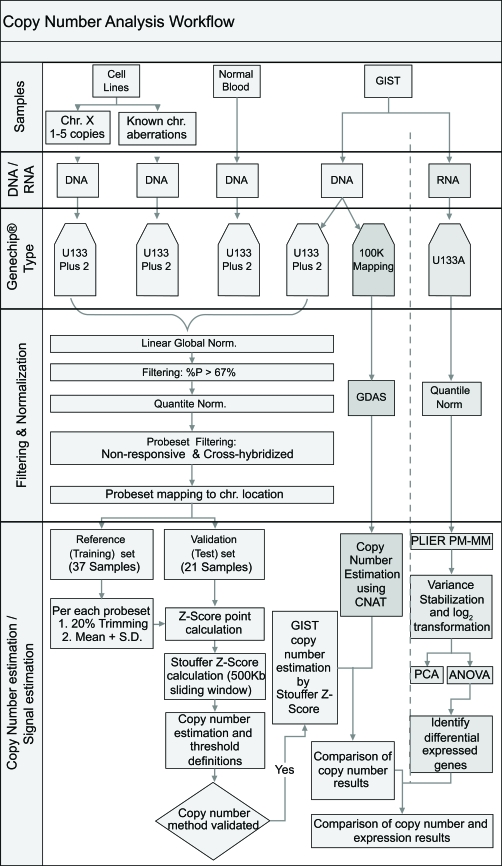
Copy number analysis workflow The steps for developing ECNA are shown here, along with the steps performed in parallel to analyze GIST copy number changes on the Mapping 100K SNP arrays and GIST RNA expression changes on U133A arrays. Details are in Supplement 2.

The copy number analysis workflow is summarized in Figure 1. The following steps were carried out sequentially: data normalization, data filtering, chromosome data mapping, reference and validation data set selection, DNA copy number estimation by computing a Z score and Stouffer Z score for each probe set, method validation and GIST samples copy number estimation. Details of the method are described in the Supplementary Materials and Methods.

### Data analysis of RNA samples hybridized to U133A arrays

Analysis was performed using Affymetrix PLIER algorithm (Affymetrix Technical Note 1)(30). Principal Component Analysis (PCA) and one-way ANOVA were performed using Partek software. Data visualization was rendered either by Partek® Genomics Suite, version 6.2 or SpotFire® DecisionSite™ version 8.1 software. One-way ANOVA analysis was used to compare the 4 GIST samples with a reference group of 6 leiomyosarcoma tumors. Data mapping between RNA expression data and DNA copy number estimation was done by matching probe set names (U133 Plus 2.0 vs. U133A array) or by corresponding chromosomal locations (U133 Plus 2.0 vs. Mapping 100K array). Each step is described in detail in the Supplementary Materials and Methods.

### 100K human mapping arrays

For data validation, genomic DNA from the same 4 GIST samples was hybridized on the 100K Human Mapping Arrays (31). The data was analyzed with Affymetrix GDAS software and the subsequent DNA copy number estimation was analyzed with Affymetrix DNA copy number tool CNAT (Affymetrix Technical Note 2) (32).

### Fluorescence in situ hybridization (FISH)

FISH was performed in 3 GIST cases, using fresh frozen touch preparations from all 3 tumors, as well as on paraffin sections for 2 of these tumors. BAC clones for PRKAR2B and PEX11B were obtained from BACPAC Resources. The PRKAR2B probe comprised two overlapping BAC clones, RP11-120N6 and RP11-258L19, labeled by nick translation with Spectrum Orange (Vysis, Abbott Laboratories, IL). A chromosome 7 centromeric plasmid probe (p7t1)(33) labeled with Spectrum Green (Vysis, Abbott Laboratories, IL) was used as reference. The PEX11B probe was a single BAC clone, RP11-315I20, labeled by nick translation with Spectrum Orange. The reference probe was a chromosome 1 centromeric plasmid probe (pSD1-1) (34) labeled with Spectrum Green. FISH was done according to standard procedures. Briefly, touch preparations were fixed in 3:1 methanol/acetic acid and then pretreated with pepsin-HCl at 37 ºC for 3 to 5 minutes, rinsed in PBS, fixed in 1% formaldehyde, then rinsed, dehydrated, and air-dried. Paraffin sections were de-waxed in xylenes, and then micro-waved in 10 mM sodium citrate (pH 6~6.5) solution for 5~10 minutes, cooled to room temperature, rinsed and dehydrated. The slides were then denatured in 70% formamide at 68 ºC for 2 to 4 minutes. Approximately 100 ng of labeled BAC DNA and 2 μg Cot-1 DNA (Invitrogen), was ethanol-precipitated, and resuspended in hybridization buffer. The probe mix was then denatured at 70 ºC for 10 minutes, followed by pre-annealing at 37 ºC for 30 minutes. The reference probe was denatured separately, without pre-annealing, and combined with the denatured reference probe on the slide for overnight incubation at 37 ºC. After standard post-hybridization washes, the slides were stained with 4’, 6-diamid-ino-2-phenylindole (DAPI) and mounted in VECTASHIELD® antifade mounting medium (Vector Laboratories). Analysis was done using a Nikon E800 epifluorescence microscope with MetaSystems Isis 3 imaging software. A minimum of 100 cells was scanned over separate regions for each slide. Image z-stacks were captured using a Zeiss Axioplan 2 motorized microscope controlled by Isis 5 software (Metasystems).

## Results

### Detection of copy number changes

**Figure 2:**
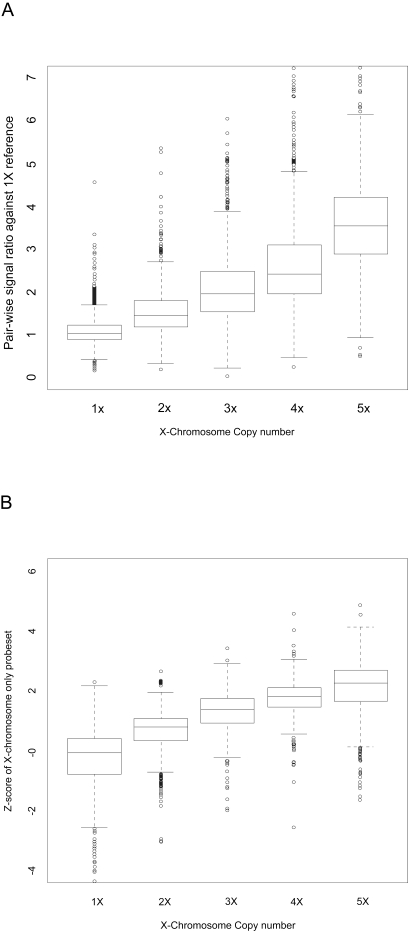
Detection of copy number changes in cell lines with variable numbers of X chromosomes The X chromosome copy number of each sample is known, which is indicated on the x-axis. The box and whisker plot (35) represents the interquartile range (between 25% and 75%) and the line within the box denotes the median. The whiskers extend to the last observation before the outliers, which are plotted individually as dots. Outliers of values greater than 7 are not plotted. **A.** The observed pair-wise signal ratio of each probe set on X-chromosome against a 1X sample is shown here. **B.** The Z-score, calculated using a reference set of 37 samples, shows similar results compared to the single sample reference in A.

To confirm that differences in signal are proportional to the differences in copy number, we performed the assay on cell lines with variable numbers of X chromosomes ranging from 1 to 5 copies. The probesets on the X chromosome show a proportional increase in signal (Fig. 2A) when each cell line is compared to a 1X cell line. The Z score, which provides a point estimation of copy number for each probeset, is derived by comparing the signal of each probeset in a sample to that of a reference sample set (Fig. 2B). Chromosomes other than the X chromosome were analyzed and found not to have copy number variation in the samples tested (data not shown).

**Figure 3:**
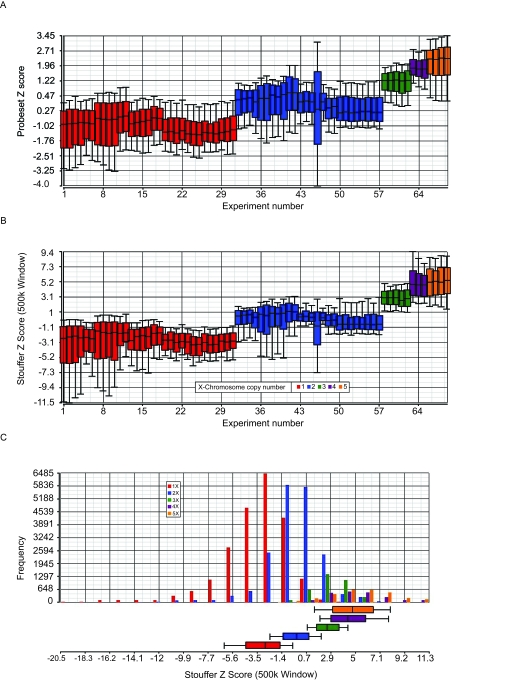

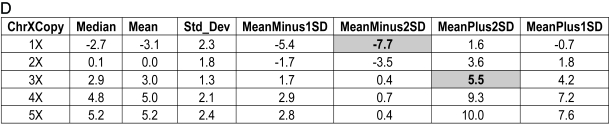
Correlation of signal with copy number The box plots are derived from analyses performed on 69 samples, described in Materials and Methods, bearing 1 to 5 copies of the X chromosome. The lower and upper whiskers are at 10% and 90% respectively. **A.** Z score. **B.** Stouffer Z score. **C.** Stouffer Z score histograms. The distribution of Stouffer Z score for these samples is represented by the boxplots below the histograms. The median as indicated in the boxplots below the 5 distribution plots is then used to assess the copy number. D. The trimmed mean of Signal and the median values for each set of samples are shown here, along with the mean +/− 2 S.**D.**s. In this study, regions with Stouffer Z scores > = 5.5, i.e. the mean Stouffer Z score for the 3X cell line + 2 S.D., are called as amplifications whereas regions with Stouffer Z scores > = −7.7, i.e. the mean Stouffer Z score for the 1X cell line −2 S.D., are called as deletions.

In chromosomal copy number estimations, a range of values is typically seen. It is important to choose thresholds or cut-off values, above or below which a region may be called amplified or deleted. The 69 samples that passed the 67% present call rate cut-off value and used in these analyses (Supplementary Table 1), have known numbers of X chromosomes (Fig. 3). In this study, Z scores in windows of 500,000 bp were used to compute Stouffer Z values. The tighter distribution seen with the Stouffer Z sliding window approach reflects the reduction of noise obtained (Figs. 3A, B) and was used for final copy number estimations. A clear separation was observed between the median Stouffer Z scores for each of the 5 sample sets, bearing 1 to 5 X-chromosome copies, and 2-fold changes could be distinguished by this method (Fig. 3C). In this model, a 2-fold change between 2X and 4X was easier to determine than the change between 1X and 2X. However, 3-fold or greater changes can be distinguished much more easily than smaller changes. When assessing copy number changes in unknown samples, it is important to use thresholds with defined levels of confidence. Since the median and mean Stouffer Z values were highly similar (Fig. 3D), in subsequent analyses we chose to use the mean values, plus or minus 2 S.D. as the threshold value to identify chromosomal deletions and amplifications. These threshold values are highlighted in Figure 3D.

### Validation of known deletions and amplifications

**Figure 4:**
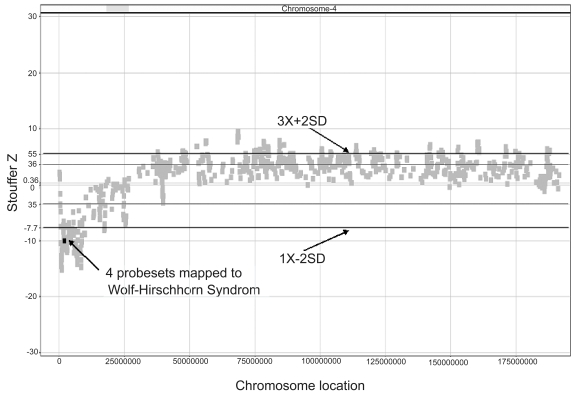
Detection of a known chromosome 4 deletion in a cell line derived from a Wolf-Hirschhorn patient 211 probesets located at the p-arm are below the line at −7.7 and hence are called as deleted. 4 of these probesets (1556651_at, 1557300_s_at, 203112_s_at and 34225_at) map to the Wolf-Hirschhorn syndrome gene, and are shown in bold.

**Figure 5:**
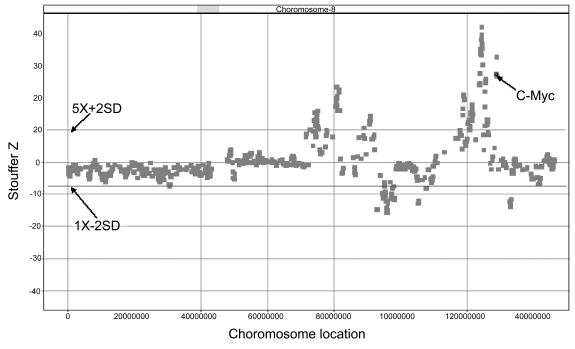
Detection of known chromosome 8 amplifications in the SK-BR-3 breast cancer cell line The probeset corresponding to the c-MYC oncogene at chromosome 8q24 is located within the amplified region and is indicated by the arrow.

Applying the cutoffs listed above, the known deletion on chromosome 4 (4p16.3) from the NA04126 cell line, derived from an individual with Wolf-Hirschorn syndrome, was detected with a number of probesets falling below the 2 S.D. line (Fig. 4). Of these, 4 probesets (shown as blue dots in the inset image) map to the *WHCR* gene, known to cause Wolf-Hirschorn syndrome. In contrast, analyzing the breast cancer cell line SK-BR-3, in which portions of chromosome 8 q are known to be amplified, a number of probesets are shown to be highly amplified, including those representing the *c-myc* oncogene (Fig. 5). *c-Myc* is commonly amplified in breast cancers and is known to be amplified in SK-BR-3. These data demonstrate that known deletions and amplifications in cell lines can be accurately detected using ECNA.

### Copy number changes in GIST

**Figure 6:**
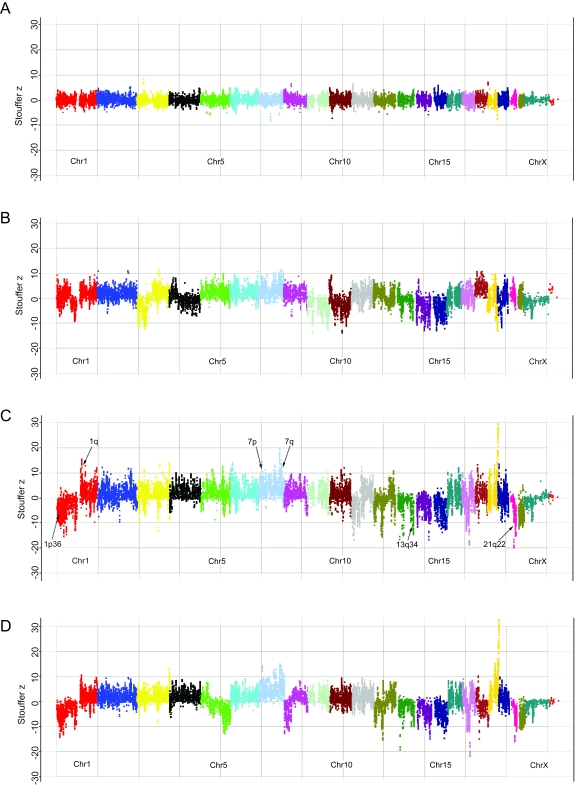
A global view of copy number changes in GIST samples Each chromosome is individually colored. The samples displayed here are Normal (**A**), GIST 198 (**B**), GIST 159 (**C**), and GIST 199 (**D**). The arrows indicate specific regions highlighted in the paper.The sample in D was obtained from the same patient as the sample in C but at a later time point (14 months), and shows additional losses at 5p and 8q.

The next step was to apply this methodology on tumor samples. GIST is an ideal tumor model for testing the sensitivity of this system, since it has relatively few copy number changes, and these are well-documented using both low and high resolution approaches (29, 36). We therefore analyzed 5 GIST genomic DNA samples for a global assessment of segmental gains and losses. Figure 6 shows the genomic view of the ECNA data for 3 of these tumors. There are chromosomal regions identified as clearly changed in all three GIST samples compared to the control sample (Fig. 6A). For example, in all tumors, the majority of probe sets in 1p are well below the 2X copy number line (0 on the y-axis.) The 1p-arm appears to fall at about a 1X copy number, indicating loss of 1p, while 1q appears to be gained in these samples.

The 2 samples (GIST#159,199) originating from the same patient from two subsequent recurrences, at 14-month intervals, showed very good concordance overall between the copy number changes (Figs. 6C and 6D, and Supplementary Table 2). Interestingly, GIST#199, the later recurrence, showed additional losses at 5q23-35 and 8p12-23 as compared to GIST#159, suggesting the possibility of deletions of candidate tumor suppressor genes involved in tumor progression.

The copy number changes detected by ECNA are listed in Supplementary Table 2. Briefly, the majority of GIST tumors showed losses of 14q (4/5 samples), 22q (3/5 samples) and 1p (5/5 samples). Furthermore, smaller regions of loss were consistently noted, such as 1p36 (seen in 4/5 GIST samples), 13q34 (4/5 samples) and 21q22 (seen in 3/5 GISTs). The two GIST samples harboring mutations in *PDGFRA* exon 18 did not show distinct findings compared to the 3 samples harboring mutations in exon 11 of the *KIT* gene. GIST#171 showed the lowest number of alterations, while GIST#204 had the most copy number changes, more similar to the samples with *KIT* mutations. A summary of our findings in comparison with other copy number analysis methods, such as CGH (1) and BAC-array CGH, for which results have been previously reported in GIST, is shown in Table 1.

**Figure 7:**
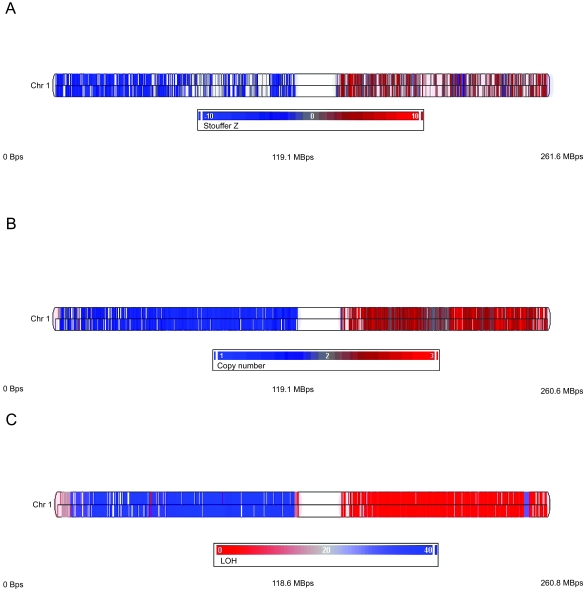
Comparison of copy number results on chromosome 1 obtained with ECNA and Mapping 100K SNP arrays Individual probesets or SNPs are represented by a vertical line mapped to chromosomal location. (top is +; bottom is −) Blank areas contain no probe-sets or SNPs. **A. ECNA copy number results.** The probesets are colored according to Stouffer Z distribution from −10 to 10. **B. 100K SNP array copy number results.** The SNPs are colored according to copy number estimation from 1 to 3 obtained using the CNAT algorithm as described in Materials and Methods. C. LOH results from **100K SNP array.**

To further validate our assay, we performed a comparison with another copy number technique with similarly high resolution. Figure 7 shows the concordance of our results on chromosome 1, with copy number analysis performed on the GeneChip Human Mapping100K arrays. Results are similar between the two methods; both reveal deletions and gains on chromosome 1p and 1q, respectively (Figs. 7A and B). The U133 Plus 2.0 arrays are gene-centric, whereas the SNP arrays span coding as well as non-coding regions of the genome. This complementarity of coverage is evident in the distribution of probesets or SNPs in the respective arrays. The SNP arrays additionally provide allele-specific information, as illustrated by the loss of heterozygosity (LOH) results in Figure 7C.

### Comparison of copy number changes with expression data and validation using FISH

**Figure 8:**
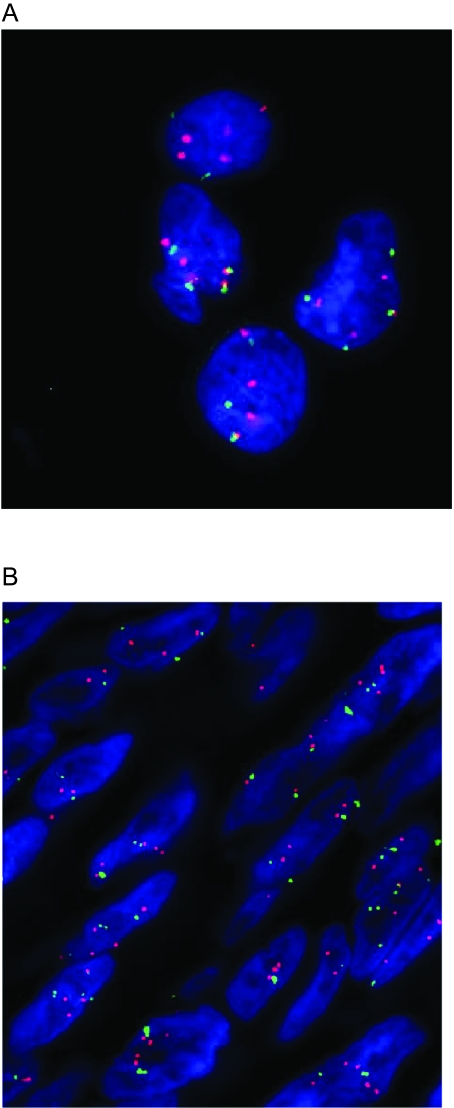
Confirmation of novel gains on chromosomes 7q22 and 1q21.2 using fluorescence in situ hybridization (FISH): A FISH for PRKAR2B on a touch preparation from GIST198. The majority of cells have 4 to 5 pairs of PRKAR2B (Red)/7 centromere (Green) signals. **B.** FISH for PEX11B on a paraffin section from GIST 199. The image is a compressed stack of 10 z-section images taken at 0.5 micron intervals, allowing display of all signals present through the depth of the tissue section. The majority of cells have 3–4 copies of PEX11B (red), as well as at least 3 copies of chromosome 1 centromeric probe (green).

One-way ANOVA identified probesets that were significantly over- or under- expressed in GIST samples compared to leiomyosarcoma tumor samples. Some regions were identified that showed both copy number change and a corresponding difference in expression as indicated by a significant p-value. Some of these low p-value probesets were mapped back to the chromosomes and regions were selected that showed both copy number change as well as significant difference in expression. Of these, two genes, *PRKAR2B* on chromosome 7 and *PEX11B* on chromosome 1 showed gains of approximately 5-fold by their respective Stouffer Z scores, and expression levels that were significantly higher in GIST compared to leiomyosarcomas. FISH analysis showed increased copies of *PRKAR2B* in the majority of the cells of GIST 198, 4–5 signals/nucleus (Fig. 8A), GIST 199, 4 signals/nucleus, and GIST 159, 2–3 signals/nucleus. However the reference centromeric chromosome 7 probe also showed increased copy number in all 3 GIST cases tested, with the *PRKAR2B* to chromosome 7 centromere ratio close to 1 (range 0.8–1.2). This result correlates with the whole chromosome 7 gains observed in ECNA (Table 1). Similar findings were found with *PEX11B* on 1q21.1, with extra copies of both *PEX11B* and chromosome 1, with a ratio close to 1. One tumor (GIST199) showed 3–4 *PEX11B* signals/nucleus (Fig. 8B) and the other (GIST159) 2–3 copies.

## Discussion

In this study we designed a method to estimate chromosomal copy number by hybridizing genomic DNA to Human Genome U133 Plus 2.0 arrays, typically used to study levels of RNA expression. An important advantage of our novel assay is that it requires only a very limited amount of DNA, i.e. as little as 5 ng starting material. Additionally, many of the advantages of an established array platform such as whole genome representation, probe set annotations and algorithms to estimate probe set signals were available to us by using this expression array approach.

We developed this method by taking samples with known differences in X-chromosome copy number. We chose a sliding window approach to generate Stouffer Z scores that were used to estimate copy number changes. The approach was then applied to and confirmed on cell lines with known chromosomal abnormalities. Finally, we used this approach to assess copy number changes in gastrointestinal stromal tumor (GIST) samples. GISTs are known to have copy number aberrations, some of which have been identified by other techniques.

In a recent study, Auer et al. (37) used a similar gene resolution analysis of copy number variation, the Affymetrix U133 Plus 2.0 arrays. Similarly, the authors conclude that this approach provides more reproducible results than custom-made BAC CGH arrays, that can be compared among different laboratories and can be combined with gene expression data using the same platform. Their results show a good concordance between the copy number changes detected by a 19k BAC high density microarray platform and the Affy expression arrays. Comparable with our approach, the authors choose various cell lines with known amplifications/deletions, such as neuroblastoma cell lines, to validate the variations in gene copy number. However they do not extend the use of this application to routine clinical tumor samples.

The most common findings, which have been reported by both conventional and BAC-array CGH, include losses of part or all chromosome 14, loss of chromosome 22, and loss of 1p (26, 29) (Table 1). Our method confirmed these results, showing a high incidence of 14q, 22q and 1p losses. Furthermore, we provide evidence that increased resolution in the current platform facilitated the identification of small alterations that were missed by a lower resolution BAC-array CGH platform. Three areas of interest were pinpointed by this method including losses of 1p36, 13q34 and 21q22, the first two previously highlighted by BAC array CGH, while the third locus being a novel finding. In addition, novel gains of *PEX11* on 1q21 and *PRKAR2B* on 7q22 were confirmed in this study via FISH and this chromosomal region was shown to have significant difference in expression patterns when compared to a control sample.

Several copy number analysis methods are now available. The amount of DNA needed varies among the different methods, from as little as 5 ng as used in this study, to 400 ng (8) or 2 μg (7). ECNA uses whole genomic DNA without complexity reduction followed by amplification with ϕ29 DNA polymerase, in contrast to the WGSA method used on SNP arrays (8, 10, 31). We analyzed the same GIST samples with the SNP array method. Despite the fact that the assay and array designs are distinct, the results obtained are highly similar (Fig. 7). While the SNP arrays have very dense genomic coverage, the HG U133 Plus 2.0 arrays are gene-centric, and so may have representation in regions where SNP coverage is limited or absent. Thus, in addition to showing good concordance, these methods are complementary. Most copy number analysis methods described thus far generate a list of genomic regions undergoing copy number alterations, without further details on their impact on gene expression. ECNA promises to link the areas of loss or gain with information related to the expression level of their corresponding probe sets, as RNA from the same sample can be used to analyze levels of RNA expression on the same platform.

Many of the advantages of an established array platform such as whole genome representation, probe set annotations and algorithms to estimate probe set signals were available to us by using this expression array approach. A clear advantage to this approach is that copy number alterations may be studied in other species, such as mouse, rat, and other model organisms for which expression arrays are available, and for which SNPs have not yet been mapped. This assay has been successfully used by other researchers to detect copy number changes on HG U133 Plus 2.0 arrays (38). Additionally, experimental evidence has shown that this assay may be used on Affymetrix tiling arrays (K. Wu, unpublished data) to assess copy number changes. We also believe that this approach will prove valuable in studying copy number aberrations in clinical samples because of the availability of relatively small amounts of starting material.

## Supplementary Material

**Data normalization:** A two-step data normalization procedure was performed, to ensure that data from different experiments are comparable on the same scale. The first step is a linear global normalization of each experiment individually through Affymetrix GCOS software. We chose a percent present call rate of 67% as a cut-off value to decide if a particular experiment should be used for downstream DNA copy number analysis. Of 79 U133 Plus 2.0 arrays that were hybridized for DNA copy number estimation, 69 passed the 67% cutoff. These 69 linearly normalized experiments were carried on to the second normalization step. A quantile normalization procedure was applied to all 69 samples together using Partek software. The second step quantile normalization makes each experiment in the set have the same distribution, while maintaining the relative rank of individual probe set within each experiment.
**Data filtering for “non-responsive” and cross-hybridizing probe sets:** AFFX bacterial spike-in control probe sets, not used in the hybridizations in this study, were used to filter out “non-responsive” probe sets. The background signal distribution of the AFFX bacterial control probe sets was examined and the signal mean + one standard deviation was used as cutoff value to filter the average signal values in 20 normal human blood DNA samples. Probe sets in the normal blood DNA sample with average signal value below this cutoff in these 20 samples were excluded from all samples, and therefore from downstream copy number estimations. Cross-hybridizing probes were removed by filtering out all probe sets with designation of “_x_”, as well as “AFFX-hum_alu_at”. After filtering, 44688 probe sets (of a total 54675 probesets on the array) remained for downstream data analysis.
**Probe set mapping to chromosome locations:** Each probe set was mapped to its corresponding chromosome location based on Affymetrix NetAFFX™ annotation (HG build 35). The mapped file was subsequently sorted by chromosome then by chromosome location. We used the middle point of the probe set selection region (PSR) as each probe set’s chromosomal coordinate.
**Reference set selection:** From the 69 quantile-normalized experiments, we partitioned the data into reference set and test set. Our reference set consists of 37 DNA samples which passed the QC parameter, and are representative of all sample types used in the study. The composition of the reference data set is shown in Table 1 (Supplement). We computed the normal distribution parameters for each probe set using a trimmed sample list for each probe set from the reference set. The top 10% and bottom 10% signal values in the training set were omitted, the remaining data values are used for computing reference set parameters.
**Z-score computation:** In order to estimate copy number, we first consider each probe set as a single data point. To estimate the single point copy number, we used Z-score, derived from the standard normal distribution of the probe set signal. The Z scores are mapped to chromosomal locations based on Affymetrix NetAffx U133 Plus 2.0 array annotation (Human Genome Build 35). All data points are sorted first by chromosome, then by chromosomal coordinates. To estimate the copy number of a region on a chromosome, we used the neighboring probe sets in a sliding window approach. We combined data points within a window using the meta-analytic technique Stouffer Z to obtain a significant window (Sutton et al. 2000). We used a sliding window size of 500,000 bp in all Stouffer Z computations in this paper.
Sutton, A., K. Abrams, D. Jones, T. Sheldon, and F. Song. 2000. *Methods for Meta-Analysis in Medical Research.* John Wiley and Sons, Ltd.

**Table S1:** Experimental samples are listed by Experiment name, sample type, number of X chromosomes, gender, percent Present calls, and representation in the Reference set. All replicates (indicated by a, b or c) were at the sample preparation level, except for the Normal Blood DNA samples which were at the array hybridization level.

Expt Name	Sample_type	Gender	ChrXCopy	% Present	Reference
1Xa	Cell_line	M	1	88.4	Y
1Xb	Cell_line	M	1	83.4	N
1Xc1	Cell_line	M	1	76.9	N
1Xc2	Cell_line	M	1	85.6	N
2Xa	Cell_line	F	2	82.2	Y
2Xb	Cell_line	F	2	86.8	Y
2Xc	Cell_line	F	2	88	Y
3Xa	Cell_line	F	3	89.2	Y
3Xb1	Cell_line	F	3	80.8	Y
3Xb2	Cell_line	F	3	88	N
3Xc1	Cell_line	F	3	78.8	N
3Xc2	Cell_line	F	3	87.5	N
4Xa	Cell_line	F	4	92.6	Y
4Xb	Cell_line	F	4	93.1	Y
4Xc	Cell_line	F	4	90.6	N
5Xa1	Cell_line	F	5	76.9	Y
5Xa2	Cell_line	F	5	84.7	Y
5Xb	Cell_line	F	5	88	N
5Xc	Cell_line	F	5	87.6	N
DelChr13a	Cell_line	F	2	86.2	Y
DelChr13b	Cell_line	F	2	85.3	Y
DelChr13c	Cell_line	F	2	87.8	Y
DelChr4a	Cell_line	F	2	67.2	Y
DelChr4a	Cell_line	M	1	86.8	N
DelChr4b	Cell_line	M	1	87.2	N
DelChr8a	Cell_line	M	1	70.2	N
DelChr8a	Cell_line	M	1	85.1	N
DelChr8b	Cell_line	M	1	83.5	N
DelChrXb	Cell_line	M	1	67.2	N
DelChrX_A	Cell_line	F	2	85.3	Y
DelChrX_B	Cell_line	F	2	88.1	Y
GIST159a	GIST	M	1	79.3	N
GIST159b	GIST	M	1	75	N
GIST171a	GIST	M	1	83.2	N
GIST171b	GIST	M	1	80.2	N
GIST198a	GIST	M	1	81.2	N
GIST198b	GIST	M	1	80.4	N
GIST199a	GIST	M	1	77.7	N
GIST199b	GIST	M	1	82.7	N
GIST204a	GIST	F	2	86.2	N
GIST204b	GIST	F	2	86.6	N
SKBR3a	Cell_line	F	2	94.5	N
SKBR3b	Cell_line	F	2	94.3	Y
Sample01A	Normal_blood_DNA	F	2	87.6	Y
Sample01B	Normal_blood_DNA	F	2	80.4	Y
Sample01C	Normal_blood_DNA	F	2	78.6	Y
Sample02a	Normal_blood_DNA	F	2	80	Y
Sample02b	Normal_blood_DNA	F	2	74.8	Y
Sample02C	Normal_blood_DNA	F	2	83.9	Y
Sample03b	Normal_blood_DNA	F	2	77.8	Y
Sample04a	Normal_blood_DNA	F	2	73.7	Y
Sample04b	Normal_blood_DNA	F	2	78.2	Y
Sample04b	Normal_blood_DNA	F	2	81.9	Y
Sample05A	Normal_blood_DNA	M	1	85	Y
Sample05B	Normal_blood_DNA	M	1	81.8	N
Sample05C	Normal_blood_DNA	M	1	82.4	N
Sample06B	Normal_blood_DNA	M	1	78.8	Y
Sample07A	Normal_blood_DNA	M	1	86.4	N
Sample07B	Normal_blood_DNA	M	1	86	Y
Sample07C	Normal_blood_DNA	M	1	87.7	N
Sample08A	Normal_blood_DNA	F	2	84.6	Y
Sample08B	Normal_blood_DNA	F	2	88.8	Y
Sample08C	Normal_blood_DNA	F	2	84.8	Y
Sample09A	Normal_blood_DNA	M	1	86.2	Y
Sample09B	Normal_blood_DNA	M	1	88.2	Y
Sample09C	Normal_blood_DNA	M	1	89	N
Sample10A	Normal_blood_DNA	M	1	85.8	Y
Sample10B	Normal_blood_DNA	M	1	87.5	N
Sample10C	Normal_blood_DNA	M	1	79.4	Y

Table S2.

## Figures and Tables

**Table 1:** 

CGH methodology	Gains	Losses	Reference
Conventional CGH	**1p, 1q**, **2q, 3q, 4, 5, 6p, 7, 8**, **9, 10, 11q**, **12q, 16, 17, 18, 19, 20**	**1p, 1q**, 2, **3p,** 4, 5p, **6q**, 7p, 8p, **9p, 9q**, **10q**, **11p**, **12, 13q**, **14, 15q, 17p**, **18q, 19q, 21, 22q**	El-Rifai, 2000
BAC array CGH	**3q**	**1p36**, **6q12**, **9p**, **13q34**, **14, 15**, 22	Granitto, 2004
ECNA*	**1p, 1q**, **2q, 3q**, **4**, **5, 6p, 7, 8, 9, 10, 11q, 16, 17, 18, 19**	**1p36**, **1q**, **3p, 9p, 9q**, **10q**, **11p**, **12**, **13q34**, **14q, 15q, 17p, 18q, 19q**, **21q22, 22q**	Present study

* Seen in ≥2 GIST tumors; bolded regions indicate that were identified by at least 2 CGH methods.
